# The Human Face and Jaws as a Danger Signal of Systemic Defect or Disorder

**Published:** 1899-09

**Authors:** J. G. Kiernan

**Affiliations:** Chicago, Ills.


### The Human Face and Jaws as a Danger Signal of Systemic Defect or Disorder.

BY J. G. KIERNAN, CHICAGO, ILLS.

Abstract of paper read before the Section of Stomatology, American Medical Association, Columbus, June, 1899.

Man is a compound organism made up of many different organs, structures and systems which have their own life albeit, subordinate to the life of the organism as a whole. These structures, organs, etc., draw upon a fixed supply of nutriment, and unless this nutriment be properly distributed through the system of checks summed up in the nervous system one organ or structure will receive more than its due proportion of nutriment. The balance kept up by the checks is disturbed when the organism becomes nervously exhausted, either from nerve tire, from general disease, or from other cause. If this exhaustion occurs at a certain period, called the periods of stress, or of involution and evolution, certain structures on which is thrown peculiarly such stress are apt to be affected, either in the direction of arrested development or of hypertrophy. Prominent among the structures which mark these periods are the jaws and the teeth, considered together. The period of the first dentition is one of these periods of stress during evolution.

The period of the second dentition is another evolutionary period of stress. The appearance of the so-called wisdom tooth marks a third period, while the disappearance of the teeth from senility is the period of involution. These conditions of nerve strain may affect the organism of the individual so that not he but his descendants show the effects. To this result are often due the irregularities of the jaws and teeth resulting from the lack of balance of the proper distribution of nutriment. The defective palates are also an expression of this strain, and not of conditions like mouth-breathing due, like the defective palates to hereditary defects, evincing itself during the evolutionary periods of stress or at birth. These irregularities are danger signals, prophesying what may happen the child of the nervously-exhausted individual unless there be proper training at the periods of stress. Training which will involve brain, nervous system, nutrition and excretion. The degeneracy which results from nervous exhaustion is a prophecy of what may be, rather than a destiny. As the dentist is among the earliest of the medical specialists to whom application is made, it comes within his power to outline a course of training and treatment which will prevent the child evincing irregularities from becoming a moral lunatic, an imbecile, paranoic, a sexual lunatic, or a victim of less forms of degeneracy. Here is a point at which the dentist is afforded an excellent opportunity to take part in the beneficent work of prophylaxis of the medical profession, which has done so much for the race. The three dentitions, as they are called appropriately by Dr. Talbot, mark three periods of systemic evolution when it is possible to affect the mental and physical development favorably.

